# Correlating Lipid Membrane Permeabilities of Imidazolium Ionic Liquids with Their Cytotoxicities on Yeast, Bacterial, and Mammalian Cells

**DOI:** 10.3390/biom9060251

**Published:** 2019-06-25

**Authors:** Kendall Cook, Katharine Tarnawsky, Alana J. Swinton, Daniel D. Yang, Alexandria S. Senetra, Gregory A. Caputo, Benjamin R. Carone, Timothy D. Vaden

**Affiliations:** 1 Department of Chemistry and Biochemistry, Rowan University, Glassboro, NJ 08028, USA; cookk3@students.rowan.edu (K.C.); tarnawskk9@students.rowan.edu (K.T.); swintonjoelle@gmail.com (A.J.S.); kaminskia0@students.rowan.edu (A.S.S.); caputo@rowan.edu (G.A.C.); 2 Department of Molecular and Cellular Biosciences, Rowan University, Glassboro, NJ 08028, USA; yangd3@students.rowan.edu (D.D.Y.); arone@rowan.edu (B.R.C.)

**Keywords:** ionic liquids, lipids, permeability, cytotoxicity, leakage assay, flow cytometry

## Abstract

Alkyl-imidazolium chloride ionic liquids (ILs) have been broadly studied for biochemical and biomedical technologies. They can permeabilize lipid bilayer membranes and have cytotoxic effects, which makes them targets for drug delivery biomaterials. We assessed the lipid-membrane permeabilities of ILs with increasing alkyl chain lengths from ethyl to octyl groups on large unilamellar vesicles using a trapped-fluorophore fluorescence lifetime-based leakage experiment. Only the most hydrophobic IL, with the octyl chain, permeabilizes vesicles, and the concentration required for permeabilization corresponds to its critical micelle concentration. To correlate the model vesicle studies with biological cells, we quantified the IL permeabilities and cytotoxicities on different cell lines including bacterial, yeast, and ovine blood cells. The IL permeabilities on vesicles strongly correlate with permeabilities and minimum inhibitory concentrations on biological cells. Despite exhibiting a broad range of lipid compositions, the ILs appear to have similar effects on the vesicles and cell membranes.

## 1. Introduction

Ionic liquids (ILs) have emerged as a versatile molecular platform for a variety of applications. These include electrochemical, industrial, and biomedical applications both at the lab and process scale. ILs have many potential applications in biochemistry and biomedical technology such as drug delivery, enzymatic reaction enhancement, and use as biomaterials. The biomass solubilization [1,2,3,4] and protein structural stabilization and destabilization mechanisms [5,6,7,8,9] of ILs in aqueous solution have been characterized extensively. These studies have primarily focused on either the molecular diversity of IL pairs and the differential effects ion pairs have on activity or the mechanisms by which different IL species disrupt protein structure/function. 

The interactions between ILs in aqueous solution and lipid bilayer membranes have guided the development of IL-based drug formulation and drug-delivery applications [10,11,12,13,14,15,16,17,18,19,20] and opened research avenues for IL-based antibiotics and antibiotic-IL combinations. There have also been several reports highlighting both experimental and computational studies on how ILs interact with lipid bilayers [21,22]. Hydrophobic ILs can insert into lipid bilayers [2,16,19,23,24,25,26,27,28,29], leading to a bilayer reorganization [23,25,30]. ILs have also been incorporated with enzyme-hydrolyzing groups, alkyl chains, and stable anions with reported antibacterial effects [31]. 

**Figure 1:**
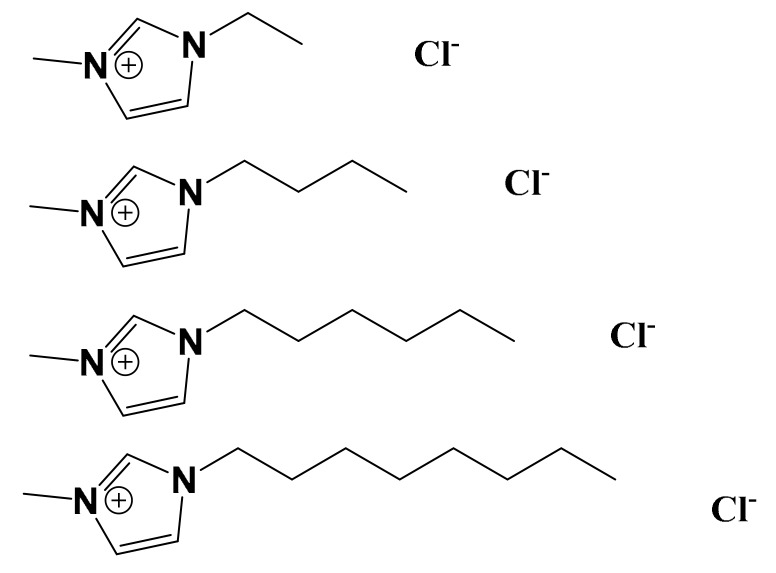
From top to bottom, [EMIM]Cl, [BMIM]Cl, [HMIM]Cl, and [OMIM]Cl.

The alkyl-imidazolium chloride ILs have been broadly studied for biomedical techniques, including membrane-permeabilization and antibacterial compounds [26,32,33]. Longer alkyl chains on IL cations can more easily embed in the bilayer, increasing their cytotoxicity [2]. The imidazolium chloride ILs discussed in this work are shown in Figure 1: 1-ethyl-3-methylimidazolium chloride, [EMIM]Cl, 1-butyl-3-methylimidazolium chloride, [BMIM]Cl, 1-hexyl-3-methylimidazolium chloride, [HMIM]Cl, and 1-octyl-3-methylimidazolium chloride, [OMIM]Cl. Increasing the alkyl chain length from [EMIM]Cl to [OMIM]Cl increases the IL hydrophobicity, and in aqueous solution, the longer-chain ILs can aggregate with critical micellar concentration (CMC) values inversely proportional to the alkyl chain length [34]. The longer-chain ILs [HMIM]Cl and [OMIM]Cl can destabilize and permeabilize membranes through surfactant effects [35].

IL cytotoxicities are correlated with cation and anion lipophilicities and related to IL-membrane permeabilization [24,36]. Studies of membrane-permeabilities and cytotoxicities of alkyl-imidazolium chloride ILs have relied on variety of model membrane systems in which lipid composition is tightly controlled and allow for fine-grained spectroscopic analysis of complex systems [21,32,37]. However, these model systems lack the diversity and complexity of natural cellular membranes in both lipid composition and membrane protein content. The varied molecular composition of cellular membranes in different organisms and species results in differential physiochemical properties of these membranes. Bacteria generally have significantly higher content of anionic lipids, no or very little sterol content, and a variable polysaccharide coat on the exterior of the cell. Additionally, there are inherent structural differences in the single-membrane architecture of Gram positive bacteria and the dual-membrane structure found in Gram negative species [38]. Fungi on the other hand have a generally zwitterionic membrane surface with higher complexity of lipid species as well as a significant variety of sterols and sphingolipids [39]. Mammalian membrane lipid compositions parallel that of fungi with differences in the sterol identities, but more importantly mammalian membranes have an added level of complexity through receptors and structures designed to allow for interaction with the extracellular matrix and signaling molecules. Differences in cellular lipid composition affect baseline permeability, bilayer fluidity, lipid raft formation, and membrane surface charges [38,39]. Furthermore, significant differences in both lipid and protein composition between membranes of bacteria, fungi, and mammals makes translating the results of the model lipid-bilayer systems to more complex cellular environments difficult.

IL biocompatibility is an essential component in developing IL-based drug delivery vehicles or any pharmaceutical or biomaterial containing ILs. Due to the essential role of membranes in cellular function, ILs that damage or disrupt the membrane have significant cytotoxic potential [37,40]. Membrane disruption can lead to loss of ion homeostasis, protein content, proton motive force, and allow entry of toxic or detrimental molecules into the cell. However, the mechanisms involved in IL mediated membrane disruption or destabilization may be impacted due to the variable membrane compositions between organisms.

Vaden and coworkers have previously reported a fluorescence lifetime-based leakage assay to quantify IL permeabilities in model lipid bilayer vesicles [11]. This assay, in which the environment-sensitive fluorescence lifetime of a fluorophore entrapped in vesicles reports on membrane leakage, is similar to the well-known calcein assay [41,42] but is very sensitive to small amounts of leakage. To understand IL membrane permeability for real cellular applications, model lipid vesicle membrane permeability results should be correlated with permeability, cytotoxicity, and antibiotic activities. In this work we correlate membrane permeabilities of alkyl-imidazolium chloride ILs with studies on a broad variety of cell lines using flow cytometry, cell-growth inhibition, and cell permeability / antibacterial assays. The results conclusively demonstrate that the lipid bilayer membrane permeabilization induced by [OMIM]Cl strongly correlates with permeabilization across a wide range of model organisms including yeast, bacterial, and mammalian red blood cells, and also with cell growth inhibition and cytoxicities.

## 2. Materials and Methods 

### 2.1. Preparation of Model DOPC:DOPG Lipid Vesicles Entrapped with Ru(bpy)_3_^2+^

1,2-Dioleoyl-sn-glycero-3-Phospho-rac-(1-glycerol) (DOPG) and 1,2-Dioleoyl-sn-glycero-3-phosphocholine (DOPC) were purchased from Avanti Polar Lipids, Inc (Alabaster, AL, USA). Cholesterol was purchased from Sigma Aldrich (St. Louis, MO, USA) and used without purification. DOPC and DOPG stock solutions were prepared with concentrations of 31.8 and 28.9 mM, respectively, in chloroform. Cholesterol stock solutions were also prepared in chloroform. Multilamellar vesicles (MLVs) were prepared by mixing 98% DOPC with 2% DOPG in chloroform and evaporating the solvent under nitrogen flow followed by further drying under vacuum for 1 h. For MLVs containing cholesterol, 28% cholesterol was added to a 70% DOPC / 2% DOPG mixture and dried. The final lipid concentration was 20 mM.

Tris (2,2’-bipyridyl) ruthenium (II) (Ru(bpy)_3_^2+^) was purchased from Fluka (St. Louis, MO, USA), and a 60 mM stock solution was prepared in HEPES buffer. HEPES buffer was purchased from Fisher. (Swedesboro, NJ, USA) Lipid vesicles with entrapped Ru(bpy)_3_^2+^ were prepared as described previously [11]. Briefly, Ru(bpy)_3_^2+^ was added to the MLVs and then subjected to a 7-cycle freeze/thaw process followed by extrusion (21 times) through two stacked 0.2 micrometer polycarbonate filter membranes in a syringe extruder (Avanti Polar Lipids, Inc). The resulting Ru(bpy)_3_^2+^-entrapped 200 nm large unilamellar vesicles (LUVs) were separated from the untrapped Ru(bpy)_3_^2+^ using a G50 Sephadex size-exclusion chromatography column with an HBS (50 mM HEPES, 50 mM NaCl pH 7) solvent. The final lipid concentration after column separation was 2 mM.

### 2.2. Fluorescence Lifetime-Based Leakage Assay of Ru(bpy)_3_^2+^-Entrapped LUVs in the Presence of ILs

As reported previously, the fluorescence lifetime of Ru(bpy)_3_^2+^ entrapped in lipid vesicles can be used to detect lipid bilayer membrane permeabilization and vesicle leakage [11]. We performed lifetime-based leakage assays in mixtures of ILs with Ru(bpy)_3_^2+^-entrapped LUVs. ILs were purchased commercially (1-ethyl-3-methylimidazolium chloride, [EMIM]Cl, Sigma; 1-butyl-3-methylimidazolium chloride, [BMIM]Cl, Sigma; 1-hexyl-3-methylimidazolium chloride, [HMIM]Cl, Alfa Aesar; and 1-octyl-3-methylimidazolium chloride, [OMIM]Cl, Alfa Aesar) and used without further purification. 1.0 M stock solutions of each IL were prepared in HEPES buffer. IL solutions were added to chromatography fractions containing LUVs entrapped with Ru(bpy)_3_^2+^. After some incubation time (~30 minutes), the Ru(bpy)_3_^2+^ fluorescence lifetimes were measured with a home-built laser-induced fluorescence (LIF) instrument, described previously [11]. Briefly, the 355 nm output of a 10-ns Continuum Minilite Nd:YAG laser excited the samples and the fluorescence emission was isolated at 610 nm with an Oriel Cornerstone 130 monochromator and detected with a photomultiplier tube. The signal was monitored with an oscilloscope. Raw data traces were fit with a single exponential decay function where the reciprocal of the decay constant is the fluorescence lifetime.

### 2.3. Minimum-Inhibitory Concentration (MIC) Assays of ILs on Bacterial and Yeast Cells

Bacteria were streaked onto Luria-Bertani (LB) agar plates from frozen stock cultures of *E. coli* D31 [43] and *S. aureus* ATCC: 27660. Growth inhibition of *E. coli* and *S. aureus* was investigated using the standard minimal inhibitory concentration (MIC) assay [44,45]. For *P. aeruginosa* we used cell line ATCC10145 and followed a literature procedure for growth [46]. Cultures of bacteria were grown by inoculating 3 mL of LB broth with a single colony from the streaked plates and allowing the culture to grow overnight at 37 °C with shaking. This culture was diluted 1:250 in fresh LB broth and allowed to grow at 37 °C with shaking until the OD_600_ was between 0.2 and 0.6. This culture was again diluted to a final density of ~10^5^ cfu/mL in LB broth. Subsequently, 90 μL of culture was added to all wells of a 96-well plate which contained 10µL appropriately diluted aliquots of ILs resulting in a final volume in each well of 100 μL. The plate was covered and incubated at 37 °C for 18 h. The OD_600_ was measured with a Spectramax M5 multimode plate reader after incubation was complete. 

### 2.4. Bacterial Membrane Permeabilization

Assays of *E. coli* outer membrane were performed similarly to as previously described [44]. A single colony of *E. coli* D31 was inoculated in LB Broth (Difco) supplemented with 100 μg/mL ampicillin and incubated in a shaking incubator at 37 °C overnight. This saturated culture was subsequently diluted 1:250 in fresh LB-ampicillin (~20 mL) and again grown at 37 °C with shaking until the OD600 was ~0.2. This culture was centrifuged at 2500 rpm for 15 min to sediment the cells. The pellet containing *E.coli* cells was then resuspended to the original volume in PBS (50 mM Na_2_HPO_4_/NaH_2_PO_4_, 150 mM NaCl pH 7) and 80 μL added to a 96-well plate containing serially diluted ILs or polymyxin B as a positive control. Immediately before the first measurement, nitrocefin was added to the plate for a final concentration of 0.25 mg/mL and mixed by pipetting, ensuring no bubble formation. The absorbance at 486 nm was measured in 5 min intervals for 90 min. All assays were performed at least in triplicate.

Inner membrane permeabilization was performed as described previously [47]. The procedure is similar to the outer membrane permeabilization assay with minor modifications. The *E. coli* were incubated overnight in plain LB media lacking ampicillin and the subsequent dilution was in LB supplemented with 5mM Isopropyl β-D-1-thiogalactopyranoside (IPTG). The experimental setup was similar with the exception that the *E. coli* were not sedimented and resuspended but were added to the plate directly in the culture medium. The positive control for the inner membrane assay was the cationic detergent cetyltrimethylammonium bromide (CTAB). The substrate for the assay was ortho-Nitrophenyl-β-galactoside (ONPG) dissolved in Z-buffer (0.1 M Na_2_HPO_4_/NaH_2_PO_4_, 10 mM KCl, 1 mM MgSO_4_, 0.05 M β-mercaptoethanol, pH 7.0) at a stock concentration of 4 mg/ml. Aliquots of this stock were added to each well (final concentration 0.6 mg/mL) immediately before measurement. The absorbance at 420 nm was measured in 5 min intervals for 90 min. All assays were performed at least in triplicate.

### 2.5. Fungal Membrane Permeabilization

*S. cerevisiae* were assayed for cell wall permeabilization as described previously [44]. Briefly, ~1 × 10^6^ cells were exposed to [EMIM]Cl, [BMIM]Cl, [HMIM]Cl, or [OMIM]Cl at 1, 0.2, 0.04, 0.008, or 0.00032 M concentrations in phosphate buffer saline (PBS) with 5 µg/mL propidium iodide (PI) for 30 min at 23 °C. Cells were washed 1X with PBS, counted in duplicate, and the fluorescence signal was evaluated with 488 nm excitation and 575 nm emission on a BD FACSCelesta instrument. Cells that are permeable take up PI and appear as fluorescent while impermeable cells do not [48]. The final percentage of cells found to be permeable for all samples was established by gating around cells on a histogram with increased PE-A signal indicated by positive control (CTAB). The positive PI control signal was established with the known permeabilization agent CTAB at 102 µM and PBS was used as a negative permeabilization control.

### 2.6. Red Blood Cell Membrane Permeabilization

Ovine red blood cells (RBCs) from defibrinated whole sheep blood was first diluted 2-fold from the stock solution in sterile PBS and RBCs were sedimented in a clinical centrifuge for 10 min. The supernatant was discarded and the pellet containing RBCs was resuspended in sterile PBS to the original volume. This was repeated three additional times such that there was no longer pink/red tint in the supernatant. The final RBC pellet was resuspended in sterile PBS to the original volume. From this suspension, 135 μL of RBCs was added to wells of a 96-well plate (round bottom wells) with each well containing serially diluted ILs or the detergent TritonX-100 as a positive control. The plate was incubated at 37 °C with gentle shaking for 60 min, then centrifuged at 4 °C to sediment the in-tact RBCs. Subsequently 6 μL of the supernatant was transferred to a new 96-well plate (flat bottom wells) containing PBS in each well for a final sample volume of 100 μL. Hemoglobin absorbance was measured at 420 nm. Percent hemolysis was calculated based on the absorbance in each well after subtraction of the absorbance in wells containing no IL, and then normalized against the absorbance in the highest concentration of the TritonX-100 positive control wells, taken to be 100% hemolysis.

## 3. Results

### 3.1. Lifetime-Based Leakage Assay Results

**Figure 2:**
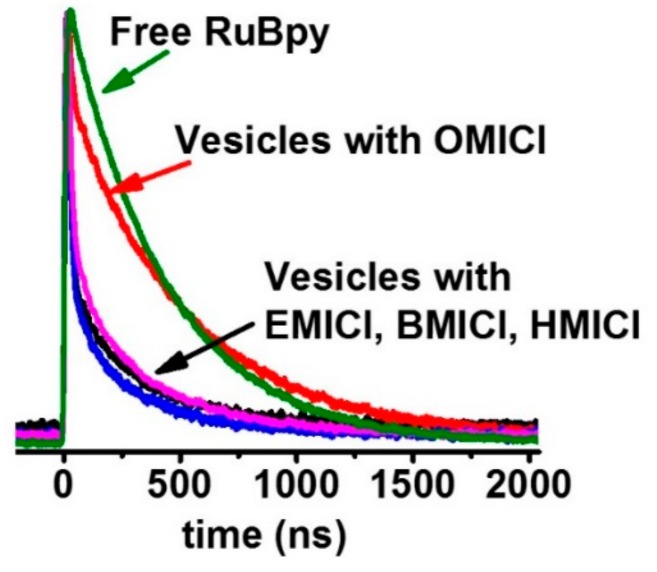
Fluorescence decay traces of 98% DOPC/2% DOPG Large Unilamellar Vesicles (LUVs) entrapped with Ru(bpy)_3_^2+^ in the presence of 0.5 M ILs.

**Figure 3:**
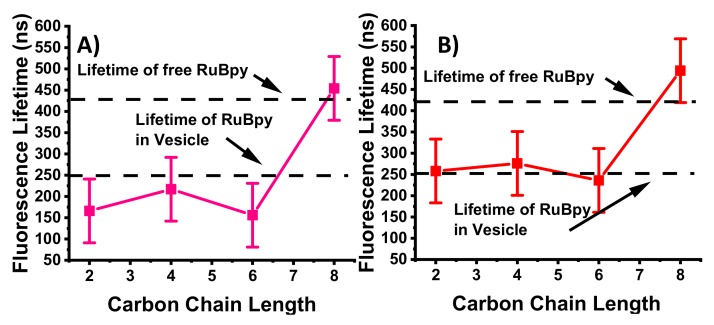
Laser-induced fluorescence (LIF0 lifetimes of LUVs entrapped with Ru(bpy)_3_^2+^ in the presence of 0.5 M ILs. (**A**) 98%DOPC/2%DOPG; (**B**) 70%DOPC/2%DOPG/28% cholesterol.

The Ru(bpy)_3_^2+^ fluorescence decay curves for the Ru(bpy)_3_^2+^-entrapped LUVs in the presence of ILs are shown in Figure 2. Previously, we have shown that the Ru(bpy)_3_^2+^ fluorescence lifetime is significantly shorter when Ru(bpy)_3_^2+^ is inside a small LUV than when it is in bulk solution [11]. When the Ru(bpy)_3_^2+^ leaks out of a permeabilized membrane, the fluorescence lifetime is similar to that of the untrapped molecule. Therefore, the decay curves clearly show that when the LUVs are mixed with [EMIM]Cl, [BMIM]Cl, and [HMIM]Cl the vesicles do not leak while the [OMIM]Cl permeabilizes the membrane resulting in Ru(bpy)_3_^2+^ leakage. This suggests that the increasing alkyl chain length, and corresponding increasing hydrophobicity, leads to lipid bilayer membrane permeabilization with 8 carbons present. This is confirmed quantitatively in Figure 3, which shows the fluorescence lifetimes plotted against increasing alkyl chain length. Averages and standard deviations are computed from multiple trials. This shows that only the [OMIM]Cl can permeabilize the LUV membranes, and this result does not change with the presence of cholesterol (Figure 3B). Please note that the Ru(bpy)_3_^2+^ fluorescence lifetime is not changed the presence of the ILs alone, and therefore lifetime changes are not due to Ru(bpy)_3_^2+^ - IL interactions.

**Figure 4:**
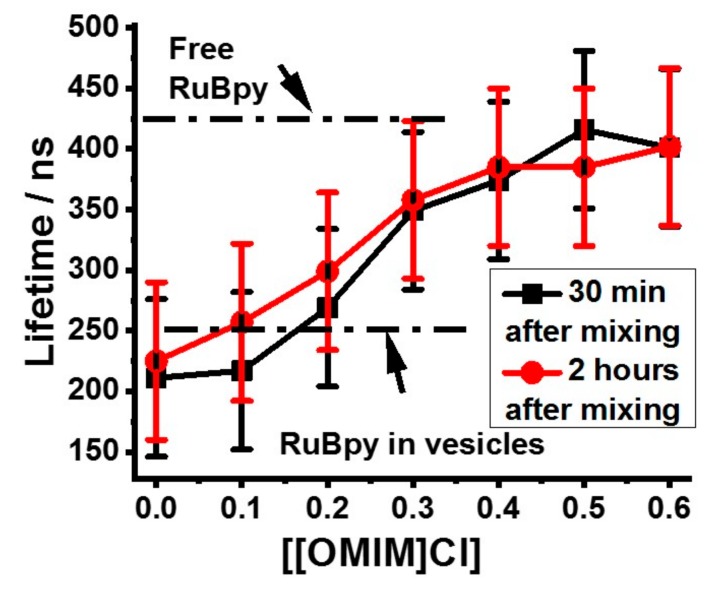
LIF lifetimes of 98%DOPC/2%DOPG LUVs entrapped with Ru(bpy)_3_^2+^ in the presence of increasing [OMIM]Cl concentrations. Data were collected within either 30 min or 2 h after mixing LUVs with ILs. Data changes likely reflect uncertainties in measured values.

The concentration-dependence of membrane permeabilization by [OMIM]Cl is shown in Figure 4, which presents vesicle-entrapped Ru(bpy)_3_^2+^ lifetimes measured at different IL concentrations. Samples were measured at different time points after mixing with ILs, but there are no significant differences between the two data sets. The inflection point in the plot in Figure 4 is at about 0.25 M IL concentration. Presumably at this point about half the vesicles have been permeabilized. Notably, this concentration matches almost exactly the CMC value of [OMIM]Cl [34]. The [OMIM]Cl IL is known to destabilize and permeabilize vesicle membranes [35], and the LIF-based leakage assay results are consistent with the results from the literature.

### 3.2. MIC Assay Results for ILs on Yeast and Bacteria

The antibacterial activity of the ILs was examined by using a broth microdilution method for determination of the minimal inhibitory concentration (MIC) values. The MIC results for [EMIM]Cl, [BMIM]Cl, [HMIM]Cl, and [OMIM]Cl against *E. coli*, *S. aureus*, and *P. aeruginosa* are shown in Table 1. The results show that antibacterial activity follows the IL chain length. Specifically, the shortest chain length, [EMIM]Cl, exhibited no antibacterial activity against any strain tested at the highest concentrations tested (100–200 mM IL). The IL activity increases (decreased MIC) with increasing alkyl chain length. [OMIM]Cl is clearly the most effective IL at preventing bacterial growth with the lowest MIC value. Notably, there were differences among the species tested, although the chain-length dependence was evident for all bacterial strains tested. 

### 3.3. Direct Permeabilization Assays of ILs on E.coli

**Figure 5:**
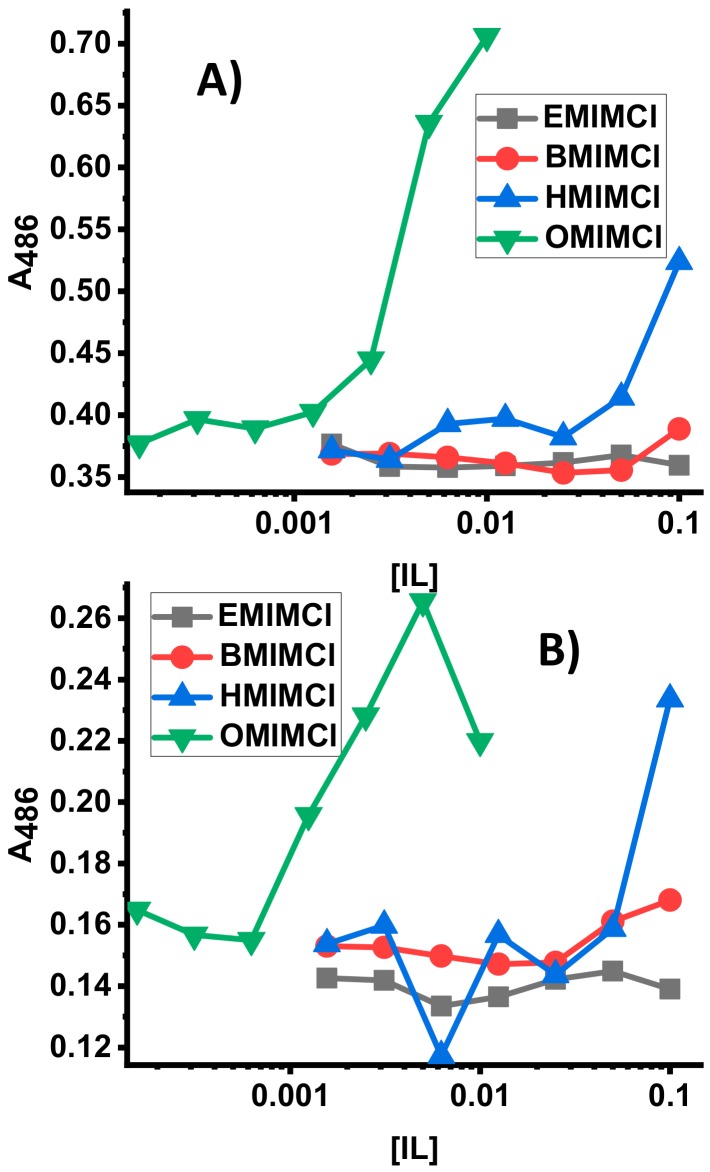
Assays of *E. coli* Membrane permeabilization by varying concentrations of ILs. (**A**) Outer membrane permeabilization, (**B**) inner membrane permeabilization. In both panels data represent absorbance measured after 30 min of exposure to ILs. All data are averages of 3 replicates.

The MIC results demonstrate that the ILs have varying degrees of efficacy as antibacterials; however these assays do not inform on the mechanim(s) of action by which the antibacterial activity is exerted. Considering the results of the vesicle leakage studies above, a permeabilization of the bacterial membrane is a potential component of the mechanism of action. *E. coli* membrane permeabilization assays were performed to investigate if the ILs caused any significant level of disruption of the bacterial membrane. Briefly, the assays rely on a bacterial enzyme and a membrane impermeable chromogenic substrate. Under normal conditions, the substrate has very low permeability across the bacterial membrane, resulting in very little conversion to the product chromophore. However, if the membrane is disrupted, the substrate has increased permeability resulting in higher degrees of conversion into the product chromophore, resulting in measurable absorbance changes. The results of these assays are shown in Figure 5. In Figure 5A permeabilization of the *E. coli* outer membrane monitored by the breakdown of nitrocefin by β-lactamase is shown, while in Figure 5B permeabilization of the *E. coli* inner membrane monitored by the breakdown of ONPG by β-galactosidase is shown. In both cases, the shortest chain ILs ([EMIM]Cl and [BMIM]Cl) exhibit very little to no detectable permeabilization at all concentrations tested. HMICl exhibits an intermediate level of permeabilization of both membranes, but only at the highest concentration test (100 mM). Finally, the [OMIM]Cl exhibited significant levels of permeabilization at 5–10 mM, paralleling the MIC results. 

### 3.4. Direct Permeabilization Assays of ILs on S. cerevisae

**Figure 6:**
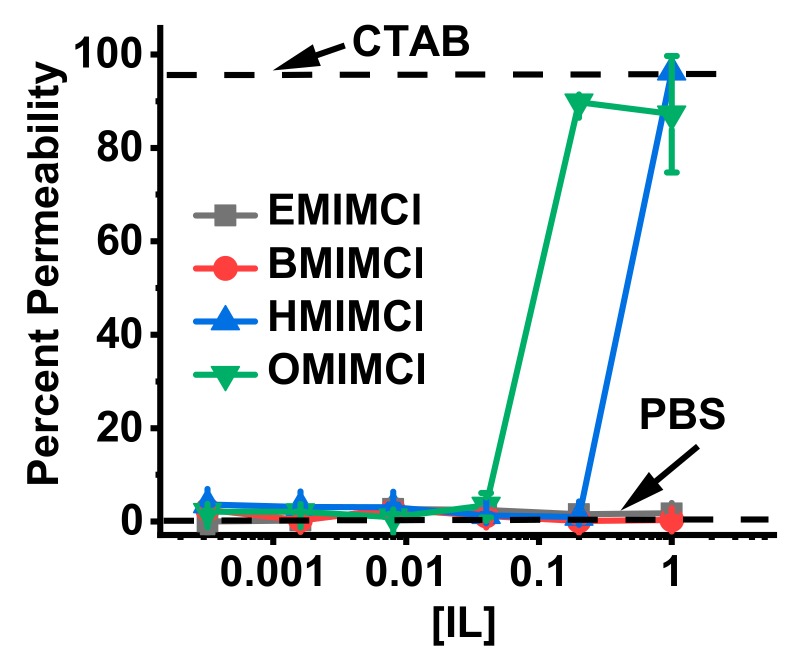
Effect of IL chain length on *S. cerevisiae* permeability. Cells with positive fluorescence at 575 nm were considered permeable. Exposure to cetyltrimethylammonium bromide (CTAB) (102 µM) yielded 94% permeable cells vs. PBS treatment 4% (dotted lines).

*S. cerevisiae* has been a widely used eukaryotic model for a variety of mammalian biological processes and disease states owing to conserved pathways and cellular structures [48]. *S. cerevisiae* has an external peptide-glycan cell wall to help protect against adverse environmental conditions [49]. Ionic liquid composition chain length has a direct and consistent effect on the ability of a given IL to permeabilize the cell wall of the yeast model organism *S. cerevisiae*. Cell permeability has classically been evaluated with the use of a cell-impermeant dye such as the fluorescent DNA binding dye, PI [48]. Living cells with an intact outer membrane are unable to take up the dye while cells which are either dead or permeable exhibit fluorescence signal Ex/Em 535/617 as evaluated by flow cytometry [50]. At all concentrations tested, short chain length [EMIM]Cl and [BMIM]Cl failed to permeabilize *S. cerevisiae* cell walls (Figure 6). At higher concentrations longer chain length ILs [HMIM]Cl and [OMIM]Cl were able to permeabilize *S. cerevisiae* with permeabilization exhibiting chain length vs. concentration dependence.

### 3.5. Direct Permeabilization Assays of ILs on Red Blood Cells

**Figure 7:**
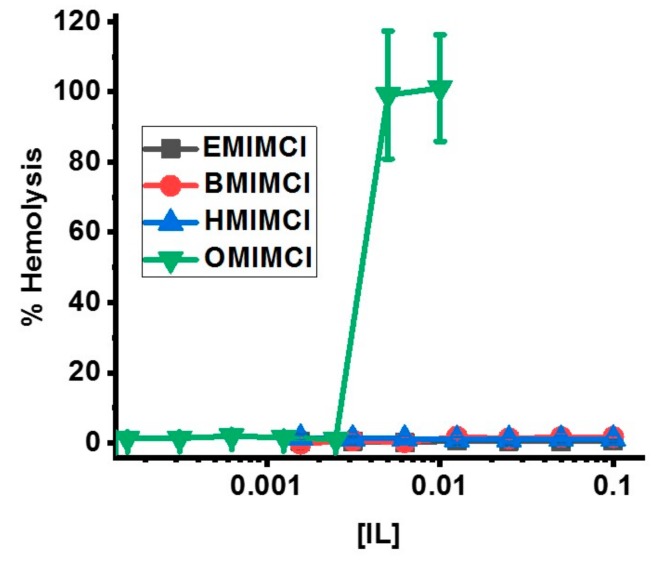
Assays of ovine red blood cell permeabilization by varying concentrations of ILs. Data represents hemolysis induced by 1-hour exposure of RBCs to ILs. Percent hemolysis was calculated by normalizing against cells treated with Triton X-100 set to 100% leakage. All data are averages of 3 replicates. Error bars represent the standard deviation. In some cases the error bars are occluded by the symbols.

While antibacterial and antifungal assays are of great importance, the potential for ILs to damage host cell membranes is a significant concern relating to potential cytotoxicity. Red blood cell (RBC) membranes are generally known to be relatively “fragile” and thus are often used as a model system for membrane-mediated cytotoxicity [51,52]. Sheep red blood cells were isolated and exposed to varying concentrations of the ILs to measure levels of released hemoglobin which is released from RBCs upon membrane disruption or damage (Figure 7). Similar to the bacterial cell membrane permeabilization assays (Figure 5), only OMICl exhibited any RBC membrane disruption at 5-10 mM. Notably, [HMIM]Cl which exhibited some bacterial membrane permeabilization at 100 mM showed no detectable RBC permeabilziation at the same concentration.

## 4. Discussion

The results of the LIF-based vesicle leakage assays, cell MIC assays, and cell leakage assays correlate the membrane-permeabilities of different alkyl methyl imidazolium ILs with their cell growth inhibition activities. This work has focused on the IL cation. The chloride is not likely involved in membrane permeability and cytotoxicity. The leakage assay clearly demonstrates that the longest-chain (and most hydrophobic) IL, [OMIM]Cl, permeabilizes model LUVs while the less hydrophobic, shorter-chain ILs do not. These results are similar to those reported in the literature using different ILs in which the cationic component was either choline, guanidine, or tetramethyl guanidine, and the anionic component contained variable alkyl chain lengths [28,53]. Recently, work from Wiedmer and coworkers also showed a strong correlation between hydrophobic content of a variety of ILs, interaction with model lipid vesicles, and cytotoxicity in mammalian and bacterial cells [54]. While none of the above referenced studies directly studied imidazolium ILs, the similarities in behavior indicate the hydrophobicity of IL components is directly linked to cytotoxic activity with both positive implications in the case of antimicrobials and negative implications in the case of host mammalian cells. This correlation has been established for other molecule types such as antimicrobial peptides and polymers [41,55] and appears to extend to ILs.

Assay results for biological cells including bacterial and yeast cell lines as well as blood cells demonstrate that the IL lipid-membrane permeabilities correlate with “real” biological activities. [OMIM]Cl at just a few mM can permeabilize both the inner and outer membranes of *E. coli* (Figure 5). [HMIM]Cl has some *E. coli* permeability (again on both inner and outer membranes) but at two orders of magnitude higher concentration. A similar conclusion can be drawn from the IL membrane-permeabilization assays on yeast cells (Figure 6) and red blood cells (Figure 7), which have significantly different membrane compositions and structures. Bacterial membranes are generally enriched in anionic lipids such as cardiolipin and those containing phosphatidylglycerol as a headgroup, yielding a net anionic charge to the membrane surface. Additionally, bacterial membranes contain high fractions of phosphatidylethanolamine (PE) headgroups which promote curvature strain and/or packing strain [56]. Gram-negative bacteria, such as the *E. coli* and *P. aeruginosa* used in this study, have two lipid bilayers with a rigid peptidoglycan layer in the intermembrane space while Gram-positive bacteria, such as *S. aureus*, have only one lipid bilayer and a rigid peptidoglycan sheet outside the cell. Both Gram-negative and -positive strains contain a polysaccharide coat. Fungi, on the other hand, have a more complex lipid bilayer composition including sterols, significant content of lipids with PE or phosphatidylcholine (PC) headgroups, sphingolipids, and glycolipids but do not contain significant anionic lipids and thus have a generally net-neutral surface charge [57,58]. The fungal cell wall which is composed of glycoproteins and carbohydrates, typically chitin and glucans, provides an additional layer of rigidity to fungal cells [59]. In general, mammalian cells are typically viewed as the most “fragile” since they are not evolved to exist in a single-celled form, and thus lack the protective extracellular structures of bacteria and fungi. Mammalian red blood cell membrane lipid composition mirrors that of fungi with high fractions of PC and sphingolipids, some PE, and sterols [60,61]. It should be noted that ovine red blood cell membranes are enriched in PE (as opposed to PC), sphingolipids, and cholesterol but are still generally net-neutral [62]. However, mammalian cells do not contain any extracellular polysaccharide wall or coat. Despite these dramatic differences in membrane compositions, the trend of IL permeabilization activity held across the three different sources tested indicating that the mechanism of permeabilization is a fundamental IL-lipid interaction, rather than a specific molecular target.

The MIC assays further help correlate IL permeability with antibacterial activities. The MIC values improve (that is, decrease) with increasing alkyl chain length. Notably, the shorter-chain ILs [BMIM]Cl and [HMIM]Cl have some MIC activity even though they have significantly lower permeabilities on the model vesicles and biological cell lines. It is also notable that MIC values are significantly lower than concentrations required for the model LUV permeabilization (200 mM for [OMIM]Cl) or yeast cell permeabilization (400 mM for [OMIM]Cl). We can directly correlate MIC with IL permeability for *E. coli*. The minimum [OMIM]Cl concentration required to permeabilize the inner and outer *E. coli* membranes is 10 mM, which is exactly the same value as the [OMIM]Cl MIC on *E. coli*. The critical value for [OMIM]Cl permeabilization is 0.2 M, which is consistent with literature values [35] and corresponds to the CMC value for [OMIM]Cl micelle formation. This strongly indicates that membrane permeability is the mechanism, or minimally a component of the mechanism, of bacterial growth inhibition and the antibacterial properties of these molecules in general.

Taken together, our results demonstrate how the antibacterial activities of alkyl imidazolium ILs are correlated with their hydrophobicities (increasing alkyl chain length) and lipid membrane permeabilities. This information will be useful for future developments of IL-based or IL-inspired drug-delivery or antibiotic formulations, perhaps involving membrane-permeable peptides [63]. It may be useful to study amino acid-based ILs (where the amino acid is anion) to modify the cytotoxicities. Previously, for special cases ILs can enhance antibiotic activities or assist in drug delivery. The current results show a proof-of-concept about how model vesicle assays can be correlated with cell-based assays to study different ILs for their biotechnological and pharmaceutical applications.

## 5. Conclusions

The results presented herein represents a parallel, side-by-side comparison of in vitro and in vivo activity of imidazolium ILs as a function of alkyl chain length. The combination of vesicle leakage and cell membrane permeabilization assays show a direct dependence on alkyl chain length, and therefore hydrophobicity, on membrane disruption and antimicrobial activity. Finally, the results show a clear parallel between IL activity against model membranes, fungal cells, bacterial cells, and mammalian red blood cells. This indicates IL hydrophobicity is a fundamental driver of membrane permeabilizing activity.

## Figures and Tables

**Table 1:** MIC values (mM) of ILs on different cell lines.

IL	*S. aureus*	*E. coli*	*P. aeruginosa*
[EMIM]Cl	>200	>200	>100
[BMIM]Cl	25	50	100
[HMIM]Cl	6.25	6.25	25
[OMIM]Cl	2.5	1.25	10
